# Characterization of PBI/Graphene Oxide Composite Membranes for the SO_2_ Depolarized Electrolysis at High Temperature

**DOI:** 10.3390/membranes12020116

**Published:** 2022-01-20

**Authors:** Sergio Diaz-Abad, Sandra Fernández-Mancebo, Manuel A. Rodrigo, Justo Lobato

**Affiliations:** Chemical Engineering Department, Enrique Costa Building, University of Castilla-La Mancha, Av. Camilo Jose Cela n 12, 13071 Ciudad Real, Spain; sergio.diazabad@uclm.es (S.D.-A.); sandra.fernandez@uclm.es (S.F.-M.); manuel.rodrigo@uclm.es (M.A.R.)

**Keywords:** green hydrogen, sulfur dioxide depolarized electrolysis, polybenzimidazole, composite, electrolysis, graphene dioxide, high-temperature

## Abstract

In this work, polybenzimidazole (PBI) membranes with different graphene oxide (GO) contents (0.5, 1.0, 2.0, and 3.0 wt %) as organic filler have been prepared. The X-ray diffraction confirms the incorporation of the filler into the polymeric membrane. The composite GO-based PBI membranes show better proton conductivity at high temperature (110–170 °C) than the pristine one. Moreover, the hydrophobicity of the PBI membranes is also improved, enhancing water management. The chemical stability demonstrates the benefit of the incorporation of GO in the PBI matrix. What is more, the composite PBI-based membranes show better phosphoric acid retention capability. For the first time, the results of the SO_2_-depolarized electrolysis for hydrogen production at high temperature (130 °C) using phosphoric acid-doped polybenzimidazole (PBI) membranes with the different GO contents are shown. The benefit of the organic filler is demonstrated, as H_2_SO_4_ production is 1.5 times higher when the membrane with a content of 1 wt % of GO is used. Moreover, three times more hydrogen is produced with the membrane containing 2 wt % of GO compared with the non-modified membrane. The obtained results are very promising and provide open research for this kind of composite membranes for green hydrogen production by the Westinghouse cycle.

## 1. Introduction

In a recent report: “Hydrogen Roadmap Europe: A sustainable pathway for the European Energy Transition” carried out by the Fuel Cells and Hydrogen 2 Joint Undertaking (FCH JU), the use of hydrogen in large quantities is highlighted to address the challenges ahead for the decarbonization of key sectors such as the gas grid, transport (particularly related to heavy duty vehicles), and industrial processes that use high-grade heat and hydrogen as chemical feedstock in Europe [1].

In addition, the electrification of the economy and the large-scale integration of intermittent renewable energy sources require large-scale energy storage systems, enabling seasonal storage and the efficient regional transport of clean energy at low cost. In this scenario, the binomial renewable energy, hydrogen, can play a paramount role for the integration of renewable energies and green hydrogen production [2].

By far, the most used and cost-effective process for the production of large amounts of hydrogen is steam reforming from fossil fuels with the issue of carbon emissions [3]. Due to this situation, different processes for green hydrogen production are proposed such as water electrolysis [4], biomass processes [5], photocatalytic water splitting [5,6], or thermochemical cycles [7,8,9]. In fact, thermochemical water splitting cycles using a high-temperature thermal renewable source have been included as one of the candidates for “green hydrogen” production in the European Union [10].

Among the different thermochemical water splitting cycles proposed for green hydrogen production, the hybrid sulfur (HyS) cycle, developed by Westinghouse Electric Company in the 1970s, is of great interest, as the theoretical voltage is 0.16 V compared with the 1.21 V for traditional water electrolysis [11]. This process comprises three main steps: (i) sulfuric acid catalytic decomposition in a high-temperature reactor to produce SO_2_ and O_2_, (ii) a SO_2_ and O_2_ separation process, and (iii) a SO_2_-depolarized electrolysis for the production of hydrogen [9,12] (Equations (1) and (2)).

(1) $$\mathrm{Anode}:\;{\;\mathrm{SO}}_{2}+2H_{2}O\;\to H_{2}{\mathrm{SO}}_{4}+2H^{+}+2e^{-}\;$$

(2) $$\mathrm{Cathode}:\;2H^{+}+2e^{-}\;\to H_{2}$$

The electrochemical step occurs in a proton exchange membrane (PEM)-based electrolyzer where the heart is, as in the case of proton exchange membrane fuel cells (PEMFCs), the membrane electrode assembly (MEA).

The most common membrane for the electrolyzer is the well-known Nafion. Nevertheless, some drawbacks arise from its use, such as limited operation temperature below 100 °C and decreased performance when exposed to high acid concentrations [13,14]. Thus, other types of membranes are being investigated to overcome the limitations of Nafion-like membranes. PBI-based membranes have demonstrated excellent behavior in PEMFCs technology when phosphoric acid is used as the doping agent [14,15]. PBI has shown excellent thermal properties with stable mechanical properties up to temperatures of 350 °C [16]. Furthermore, phosphoric acid-doped PBI membranes have demonstrated great thermal and chemical stability up to temperatures of 200 °C [17]. Therefore, PBI-based membranes will be tested in this application at temperatures higher than 100 °C, as these high temperatures will lead to higher overall efficiencies in the hybrid sulfur cycle [18].

PBI membranes have been modified in order to improve their performance in PEMFCs [19,20,21]. One of the recent trends is the addition of 2D carbon-based materials to the polymeric structure of PBI-based membranes for PEMFC technology [22,23,24]. In particular, graphene oxide (GO) and its derivatives have attracted much attention due to their features such as being two-dimensional structures with large surface area and the large number of oxygen functional groups that can potentially increase the conductivity of the polymer matrix [25,26]. What is more, membranes prepared with GO have reported improved mechanical properties [22].

So far, PBI membranes have been tested for the SO_2_-depolarized electrolysis [27,28]. However, the influence of the addition of organic or inorganic fillers has not been yet studied for the enhancement of the SO_2_ depolarized electrolysis. 

Owing to the promising performance and behavior of composite PBI membranes in other applications such as fuel cells, and particularly the promising results of PBI/GO composite membranes in fuel cell applications, in this work PBI/GO composite membranes were prepared for the SO_2_-depolarized electrolysis. Hence, the aim of this work is the preparation, characterization, and the SO_2_ electrolysis performance evaluation of phosphoric acid-doped composite PBI-based membranes with different contents of GO at high temperature. 

## 2. Materials and Methods

### 2.1. Materials

PBI solution was purchased from PBI Performance Products (abbreviation of the county, Charlotte, NC, USA) with a PBI concentration of 26 wt % with N,N-dimethylacetamide (DMAc) as solvent and stabilized with LiCl. DMAc was received from Panreac (Barcelona, Spain). H_3_PO_4_ (85 wt %) was received from Merk (Darmstadt, Germany). Graphene oxide (GO) particles obtained from graphene nanofibers (<38 µm) were kindly provided by Grupo Antolín S.A. (Burgos, Spain). All materials were used with no further purification. 

### 2.2. Membrane Preparation

Composite GO-based PBI membranes were prepared with different contents of GO (0.5, 1.0, 2.0, and 3.0 wt %) by the solvent-casting method as follows. The right amount of GO particles, for each composite membrane, was dispersed in DMA for 15 min in an ultrasound bath to obtain a homogeneous dispersion. Meanwhile, the commercial 26 wt % PBI solution was diluted by adding DMAc to reach a final concentration of 2 wt %. Afterwards, the particles were added to the diluted PBI. This solution was homogenized in the ultrasound bath for 2 h, obtaining a homogeneous black solution. The membranes were finally obtained by pouring the solution into a plate of 13 cm of diameter and evaporating the solvent in an oven at 80 °C for 24 h. Once this time elapsed, the plate was immersed in DI water to detach the membrane from the plate, as reported elsewhere [29,30]. The obtained membranes were washed in boiling water for 2 h and dried again at 80 °C for one day before their use. Similar GO–PBI composite membranes preparation was followed by other authors [24,31]. 

### 2.3. Chemical and Physicochemical Membrane Characterization

Composite membranes with different graphene oxide contents were analyzed by X-ray Diffraction in a Philips X’Pert MPD (PANALYTICAL, Malvern, UK) diffractometer applying Kα corresponding to the transition from copper radiation (λ = 1.5404 Å) using a 4 cm^2^ sample. XRD analyses were carried out recording the 2Ɵ angular region from 10° to 100° (scan rate 0.02°·s^−1^).

The morphology of the membrane surfaces was observed by using a Microscope Gemini SEM 500 field emission. To prepare the samples, all the membranes were sputter-coated with a 2 nm gold layer.

The in-plain conductivity was measured by a four-point system, as described elsewhere [29]. The sample (6.0 cm × 1.0 cm) is placed on the top of the plate, and the wires are placed on the top of the membrane, which are connected to a galvanostat/potentiostat (AutoLab PGSTAT204, Utrecht, The Netherlands) equipped with a frequency response analysis module. Experimental measurements were carried out after the membranes reached the desired temperature for at least 1 h under dry conditions. Electrochemical impedance spectroscopy (frequency range 100–10,000 Hz and amplitude of 10 mV) was used to calculate the resistance to the ionic flux (R_Ω_). Equation (3) was used to calculate the ionic conductivity, where R_Ω_ (ohm) is the value of resistance to the ionic flux, l (cm) is the distance between the wires where the potential difference is measured (1 cm), and S (cm^2^) is the transversal section of the membrane.

(3) $$\sigma \;\left(\frac{S}{\mathrm{cm}}\right)=\frac{1}{R_{\Omega }}\cdot \frac{l}{S}$$

The acid-doping level (ADL, mol H_3_PO_4_ · r.u.PBI^−1^) of the membranes was measured by immersing samples with dimensions of 2.0 cm × 2.0 cm into 85% H_3_PO_4_. Before doping, the samples were dried overnight at 80 °C to remove the humidity in the membrane (m_dryPBI_). Afterwards, the membranes were weighted and then submerged in phosphoric acid for 24 h at 80 °C. Once the wet weight of the doped PBI membranes remained constant, they were dried again to remove the absorbed water. Then, the membranes were weighed (m_dopedPBI_) again to calculate the acid uptake using Equation (4).

(4) $$\mathrm{ADL}=\frac{\left(m_{\mathrm{dopedPBI}}-{\;m}_{\mathrm{dryPBI}}\right)\cdot 98\frac{g}{{\mathrm{mol}}_{H_{3}{\mathrm{PO}}_{4}}}}{\frac{m_{\mathrm{dryPBI}}}{308\frac{g}{r.u.\;\mathrm{PBI}}}}$$

The capability of the membranes to retain the absorbed phosphoric acid was measured by the following protocol. Doped samples (2.0 cm × 2.0 cm) were immersed in a flask with deionized water at 80 °C for one hour while magnetically stirring. Then, liquid samples (1 mL) were taken each 10 min for one hour. Phosphates in those liquid samples were determined by ionic chromatography. In order to calculate the acid retention, the phosphates concentration from the last sample was used. Equation (5) was used to determine acid retention.

(5) $$\;\mathrm{Acid}\;\mathrm{retention}=\left(1-\frac{\left[H_{3}{\mathrm{PO}}_{4}\right]\cdot V}{m_{\mathrm{dopedPBI}}-{\;m}_{\mathrm{dryPBI}}}\right)*100$$

where [H_3_PO_4_] is the concentration of phosphoric acid in the water (mg mL^−1^), V is the volume of employed water (100 mL), and mdry and mdoped are the weight of the membrane before and after the doping treatment (mg), respectively.

The hydrophobicity of the membranes was determined by measuring the contact angle of a water drop in the surface of each un-doped membrane. Briefly, a water drop is dropped in the membrane surface, and after waiting 15 min for stabilization, a photograph is taken. Then, the contact angle between the water drop and the membrane surface is obtained.

The chemical stability of the membranes was measured by performing accelerated oxidation tests with the sulfate radical as oxidizing agent (SO_4_^−^·). The selection of this test was due to its very high oxidation power of this radical, which is more likely to be formed in the electrochemical reactor due to side reactions at high temperature [25,32] in the acidic sulfur environment. For the persulfate test, dried PBI-based membranes (45 mg) were introduced in a flask with an initial concentration of Na_2_S_2_O_8_ (Sigma Aldrich, St. Louis, MO, USA) of 500 ppm in 1 M H_2_SO_4_. The experiments were carried out during 8 h at 80 °C to decompose the persulfate to the sulfate radical according to Equation (6).

(6) $${\;S}_{2}O_{8}^{2-}\;\to {2\mathrm{SO}}_{4}^{-}$$

### 2.4. SO_2_ Electrolysis Tests

The electrochemical performance of the PEM reactor was studied with the standard and the PBI–GO composite membranes. The measurements were carried out in an electrolyzer with an active area of 25 cm^2^. Both anode and cathode were prepared following the same procedure. A catalyst ink prepared with commercial Pt/Vulcan (40 wt % of Pt, Fuel Cell Store, College Station, TX, USA), DMAc as solvent, and PBI as ionomer was sprayed with an air-gun until a catalyst loading of 0.7 mgPt·cm^−2^ was reached for both anode and cathode. H23C2 GDL was used as the gas diffusion and supporting layer (Freudenberg, Germany) for the anode and the cathode electrodes. The experimental set-up is shown in Figure S1. The operating procedure consisted of mixing, in stoichiometric conditions, the gas flow of SO_2_ (70 mL·min^−1^ SO_2_) (Carburos Metálicos, Spain) with Milli-Q water (0.1 mL·min^−1^). This mixture was heated to 110 °C to evaporate the liquid water before entering the anode of the cell. In the cathode of the cell, a mixture of nitrogen (100 mL·min^−1^) and steam (0.5 mL·min^−1^ of Milli-Q water vaporized before entering the cell) was introduced. The anode mixture was pre-heated at 110 °C and only fed after the reactor reached 110 °C to prevent sulfur dioxide crossover through the membrane. After reaching this temperature and introducing both reactants, the electrolyzer operated at 0.6 V for 90 min for activation at 110 °C. Afterwards, the temperature in the reactor was increased to 130 °C and electrochemically characterized.

Then, sulfuric acid was measured by collecting the anode outlet at 0.60 V, which was measured by ionic chromatography. The system was electrochemically characterized by performing linear sweep voltammetries (LSVs). LSVs were performed from 0.0 to 1.0 V at a scan rate of 10 mV·s^−1^ using a galvanostat/potentiostat (AutoLab PGSTAT204, The Netherlands).

The cathode outlet was characterized by gas chromatography with a GC-2030 (Shimadzu, Japan) equipped with a Rxi-1ms column (L = 30 m; ID = 0.32 mm; DF = 0.50 µm) for sulfurous compounds (H_2_S and SO_2_) and a Rt-Msieve 5A column (L = 30 m; ID = 0.32 mm; DF = 30 µm) for the identification of small gas molecules (H_2_, N_2_, and O_2_).

## 3. Results

**Figure 1:**
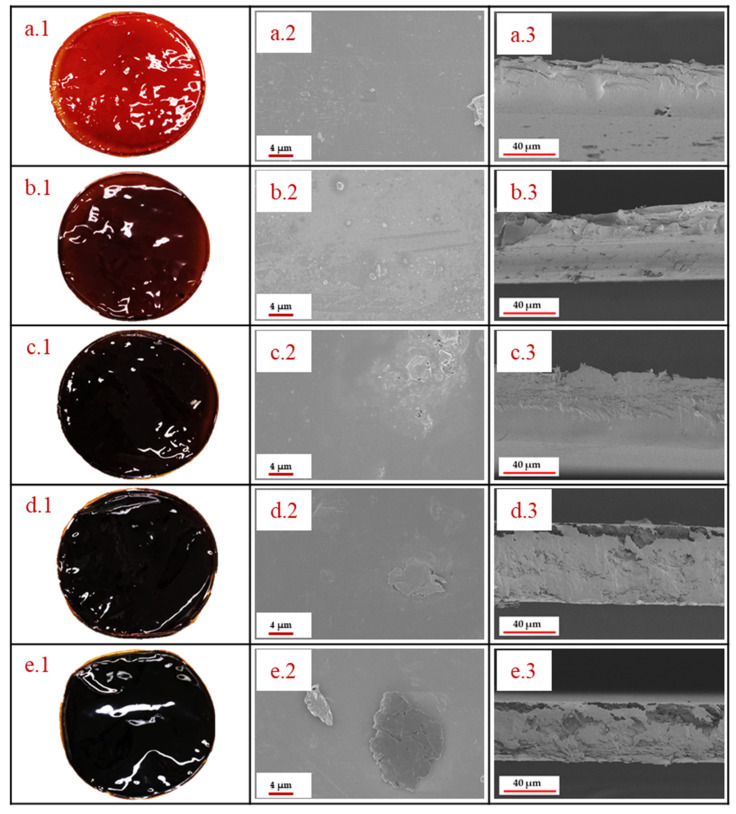
Photographs and SEM images of the PBI membranes: (**a**.1–**a**.3) Standard PBI membrane, (**b**.1–**b**.3) 0.5% GO–PBI membrane, (**c**.1–**c**.3) 1% GO–PBI membrane, (**d**.1–**d**.3) 2% GO–PBI membrane, (**e**.1–**e**.3) 3% GO–PBI membrane.

The morphology of the standard and the PBI/graphene oxide composite membranes is shown in Figure 1. GO contents were studied up to 3 wt % due to very fragile membranes obtained for higher contents of this organic filler, which would not be adequate for electrolysis operation in a PEMFC. The taken photographs show that GO has homogeneously been dispersed in the membrane, observing that the membrane became darker when increasing the amount of graphene oxide in the PBI matrix. However, all of them looked homogeneous with no visible agglomeration of GO when the amount increased from 0.5 wt % to 3 wt %, demonstrating a good dispersion and that the preparation procedure was adequate to obtain these membranes. In order to observe in detail the structure of the composite membranes, SEM images of the PBI-based membranes prepared in this work are also shown in Figure 1. It can be observed that the standard membrane (Figure 1(a.2)) has a smooth surface, demonstrating that membranes using the solvent-casting method are properly obtained. When graphene oxide is added to the membrane, almost no difference is observed in the SEM image of the 0.5 wt % membrane due to the low content (Figure 1(b.2)). For higher GO contents, some laminar structures can be observed on the surface of the PBI membranes (Figure 1(c.2,d.2,e.2,e.2)). However, those structures are covered by PBI, and there are no signs of fractures in the membrane, even for the case of the 3 wt % PBI/GO membrane, which was the highest concentration of GO. This indicates a strong interfacial adhesion between the particles and the polymer. This effect is explained due to polar/H-bonding interactions of oxygen functional groups on GO with benzimidazole groups in the PBI backbone. In this regard, acid–base interactions between PBI (pKa = 5.5) and -OH functional groups (pKa = 9.8) in GO are also responsible for a good GO dispersion in the PBI matrix [33,34]. Furthermore, π−π interactions of PBI and GO also help to maintain a homogeneous structure [35]. Similar structures and results are obtained by Dey [33] when they prepared a PBI membrane with even higher composite loading of graphene oxide. Cross-sectional SEM images are also reported (Figure 1(a.3,b.3,c.3,d.3,e.3)), demonstrating satisfactory GO introduction in the membrane up to filler contents of 2 wt %. The standard membrane shows a smooth cross-section, which showed almost no difference when an amount of 0.5 wt % GO particles was added due to the low concentration. For higher filler concentrations (1, 2 and 3 wt %), laminar structures can be observed within the inner structure of the membrane. For the case of the 1 wt % membrane, a homogeneous cross-section is obtained, but when the concentration for GO in the membrane increased to 2 wt %, small voids appeared in the part of the membrane closer to the air side. However, the structure seems to be compact and homogeneous. On the other hand, for the 3 wt % GO membrane, those voids are more evident. In this case, a layer of the GO sheets can be observed in the bottom, while the formed voids occupy most of the section close to the air side of the membrane. Furthermore, it is interesting to see that in the case of 2 wt % and 3 wt %, although some voids appear, a dense skin is formed on the surface of the membrane.

**Figure 2:**
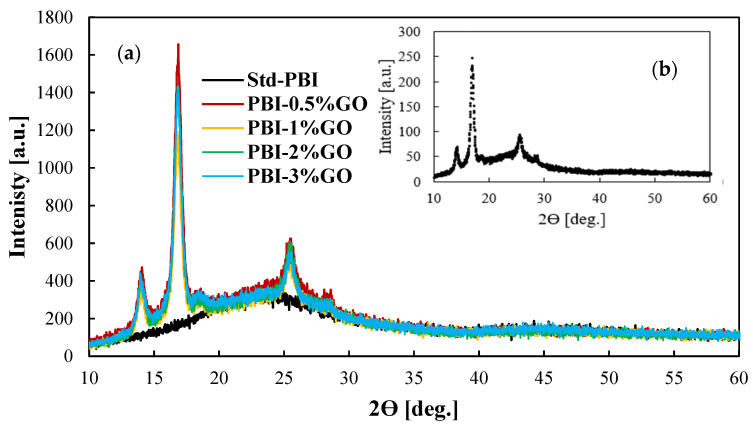
(**a**) XRD patterns of GO composite PBI-based membranes with different content of GO; (**b**) XRD pattern for the GO particles.

Figure 2 shows the XRD patterns for the prepared membranes and the GO for comparison purposes. The black line, which represents the results for the standard membrane, shows the characteristic wide peak from 20 to 30 degrees of PBI [36,37]. The inset of the figure shows the XRD pattern of the graphene oxide particles. For the composite membranes, the incorporation of graphene oxide in the PBI matrix is clear, as the same peaks obtained for the provided graphene oxide appear in the XRD patterns of the membranes. The peak obtained at 25.5° is the characteristic peak of tubular graphitic structures [38]. The peaks at 13.9° and 16.8° are related to the oxidation procedure [39]. The FTIR spectra reported in Figure S2 show the typical PBI spectra with the broad peak between 2000 and 3600 cm^−1^ due to the stretching vibrations of the hydrogen bonding. Other characteristics peaks at wavelengths of 1612 cm^−1^ (C=C), 1532 cm^−1^ (C=N), and 1438 cm^−1^ (C-N) are visible for all the membranes. However, this characterization technique does not indicate the incorporation of GO because of its low content [40].

Table 1 shows the effect of the doping step on the prepared membranes. Regarding the thickness change of the membranes, similar values were obtained for all the membranes, reaching values in accordance with others found in the literature [41,42]. ADL reaches a maximum for the 1 wt % PBI/GO membrane, being 12.5% higher than the one obtained for the standard membrane. All the membranes with graphene oxide show higher ADL values because of the interactions of oxygen functional groups of the GO particles providing extra sites to phosphoric acid to be attached [24]. The ADL value decreases from a GO concentration in the membrane of 1 wt % to 3 wt %. In this case, the effect of lowering the free volume in the membrane when increasing the amount of filler prevails over the increase in phosphoric acid receptor sites, as it is also reported by Üregen et al. [31]. In accordance with the doping level, the increase in the thickness of the membranes reaches a maximum for the membrane with 1 wt % of GO; those values are similar to others obtained for pristine PBI membranes [41,43]. Moreover, as in the case of the use of phosphoric acid-doped PBI membranes for high-temperature PEMFCs, high acid retention capability is required to be applied also for the SO_2_ depolarized electrolysis. Thus, the phosphoric acid retention capability was evaluated. The obtained values are also shown in Table 1. It can be observed that the addition of GO up to 3 wt % contributes to an enhancement of the acid retention due to more interactions between basic functional groups of GO and the phosphoric acid. 

**Figure 3:**
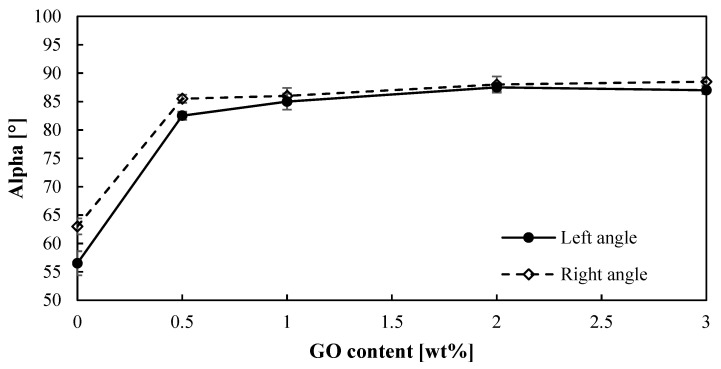
Contact angles (right and left) for the PBI-based membranes prepared.

For the hydrogen production by means of the sulfur dioxide depolarized electrolysis, the hydrophobicity of the membrane is an important feature to be considered, since water plays a key role. SO_2_ crossover due to water transport can lead to sulfur formation in the cathode, which will poison the catalyst [44]. Therefore, improving water management can reduce this crossover. Thus, the angle contacts (right and left) of a water drop onto the surface of PBI–GO membranes were measured, and Figure 3 shows the obtained results. First of all, it can be observed that both angles are very similar, which is indicative of the homogeneity of the membrane surfaces. On the other hand, the contact angle of the membranes clearly increases (almost 40% higher) when just 0.5% of GO is added to the PBI membrane when compared with the standard membrane. The same result is obtained for the other composite membranes with no effect or difference on the contact angle when the amount of GO is increased. In terms of hydrophobicity, a contact angle lower than 10° indicates a super-hydrophilic material, hydrophilic materials have contact angles between 10° and 90°, and hydrophobic materials have larger contact angles [45]. This means that when graphene oxide is added to the PBI membrane, the behavior toward water changes from hydrophilic to almost a hydrophobic material. This effect can be attributed to the hydrophobic properties of GO and an increase in the roughness of the surface of the membrane. Other works using GO to modify different materials have shown similar behavior when GO is added [46,47]. This hydrophobic effect might minimize the water transport through the membrane, thus reducing SO_2_ crossover.

**Figure 4:**
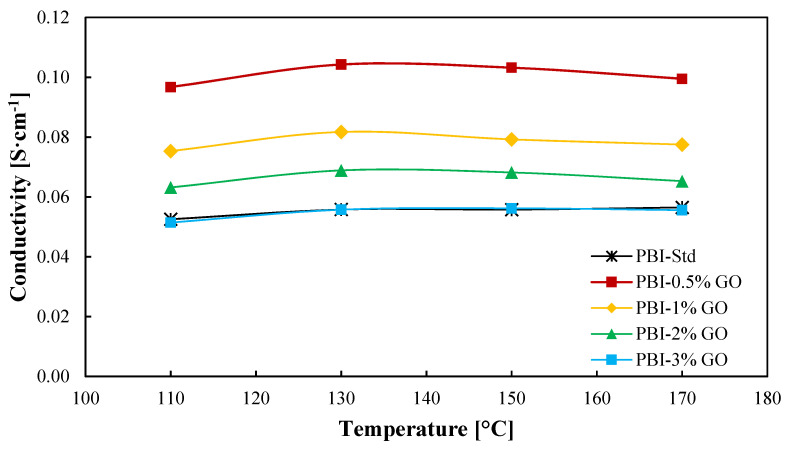
Ionic conductivity as a function of temperature of PBI and GO composite-based PBI membranes. Black line: Standard PBI; Red line: PBI–0.5%GO; Yellow line: PBI–1%GO; Green line: PBI–2%GO; Blue line: PBI–3%GO.

In-plane conductivity results are shown in Figure 4. As observed, the proton conductivity reaches a maximum for all the membranes at a temperature of 130 °C. Nevertheless, the temperature does not have a significant impact on the conductivity of the membranes. Measurements were carried out under dry conditions; therefore, at temperatures above 130 °C, acid demineralization occurs due to the loss of water [48], which is more drastic al low humidity conditions. Aili et al. [49] obtained slightly lower conductivities for a standard PBI membrane with an ADL of 10.2, reaching a maximum of 4 × 10^−2^ S cm^−1^ at 150 °C. Regarding the addition of graphene oxide to the membrane, a large improvement is observed when an amount of 0.5 wt % of GO is added to the membrane in comparison to the standard membrane. An enhancement of 86% in proton conductivity at 130 °C is obtained for this membrane in comparison with the standard membrane. From this point, the graphene oxide content causes a decrease in proton conductivity but always improving the result obtained for the standard PBI membrane until a content of 3 wt % is employed, which is when the conductivity of the composite membrane decreases to almost the same value of the standard membrane. Therefore, the increase in ADL due to GO (Table 1) partially explains the proton conductivity results, as greater ADL will lead to larger conductivities. However, this value does not explain why the 1 wt % composite membrane shows lower proton conductivity. An excess of GO can lead to a loss of conducting channels and a more tortuous inner structure, which complicates proton transport [31]. Kim et al. [24] studied films of PBI with imidazole-functionalized graphene oxide as filler. In their work, a similar result was obtained, the conductivity of the membranes increased with a filler content (graphene oxide) of 0.5% compared with the standard membrane and decreased when the amount of filler was 1 wt %. In the case of Üregen et al. [31], higher graphene oxide concentrations in the PBI matrix were used but with similar results. In their work, the proton conductivity of the membrane with a 2 wt % content of GO was higher than the standard membrane, but when the organic filler content was 5%, the proton conductivity decreased, which was also attributed to the loss of proton transport channels. 

Considering that the environment in the anode side will be very corrosive, as sulfuric acid at high temperature will be presented, the chemical stability of the PBI membranes prepared in this work was assessed. Figure S3 shows the chemical stability test performed for the prepared membranes with persulfates as an oxidizing agent. The graph shows the time at which the membranes broke in the persulfate solution at 80 °C. As observed, in all the cases, the incorporation of graphene oxide into the PBI matrix increases the chemical stability of the membranes. The optimum in GO content is observed to be 2 wt % of GO with a breakage time of 36 h, which is closely followed by the composite membrane with a GO content of 1 wt %. The chemical stability is enhanced due to the repulsion effect of negatively charged functional groups of GO and sulfate radicals. 

**Figure 5:**
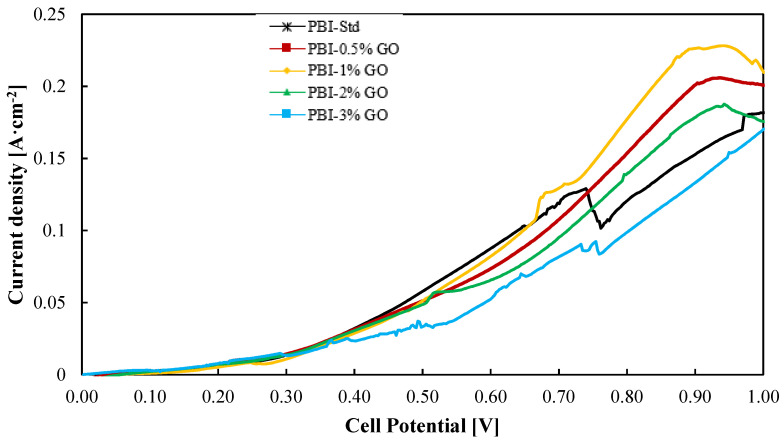
Current–voltage curves from a SO_2_ electrolyzer using the different PBI-based membranes prepared in this work at 130 °C. Potential range of 0–1 V at a scan rate of 10 mV·s^−1^. Black line: Standard PBI; Red line: PBI–0.5%GO; Yellow line: PBI–1%GO; Green line: PBI–2%GO; Blue line: PBI–3%GO.

On the other hand, most of the work published for composite PBI membranes are ex situ characterizations or HTPEM-FC tests. In this work, not only the PBI–GO composite membranes were prepared and characterized ex situ, but their use was evaluated for the SO_2_ depolarized electrolysis in an electrolyzer at bench scale (25 cm^2^). Thus, Figure 5 shows the current–voltage (i–v) curves for all the PBI–GO composite membranes and the standard one at 130 °C. The electrolyzer operated with a humidified cathode consisting of nitrogen and steam and a SO_2_ gas feed in the anode compartment where it reacts with steam. Both anode reactants are mixed before entering the cell. It can be seen that at cell voltages lower than 0.6 V, the standard membrane and the composite PBI membrane with a content of 1 wt % perform in a similar way, which is followed by the other three membranes, being the composite membrane with the highest content (PBI–3 wt% GO) the one that showed the lowest performance. At voltages higher than 0.6 V, around 0.8 V, the membrane with the best result (0.18 A·cm^−2^ at 0.8 V) is the one with 1 wt % of GO, while the standard PBI membrane shows the second worst result, 0.12 A·cm^−2^, which means 33% lower performance.

At high voltages, the reaction takes place in a major extent; hence, more transfer limitations can appear, and also some side reactions could occur at the anode and cathode due to SO_2_ crossover. At high current densities (high voltages), the standard PBI membrane performed worst because of its higher affinity with water, which will lead to higher water transport and thus higher SO_2_ crossover [44]. The lower affinity of PBI/GO membranes toward water can reduce this crossover and parasitic reactions. Parasitic reactions could be the explanation for the current density drop for the standard membrane at the cell potential of 0.72 V. In this case, due to the expected higher water transport from the anode to the cathode, the effect of side reactions will be more critical. 

PBI-based membranes have been previously tested for this application, although in different conditions and smaller electrolyzers. Table S1 summarizes some results for SO_2_-depolarized electrolyzers serving as comparison for this work. 

**Figure 6:**
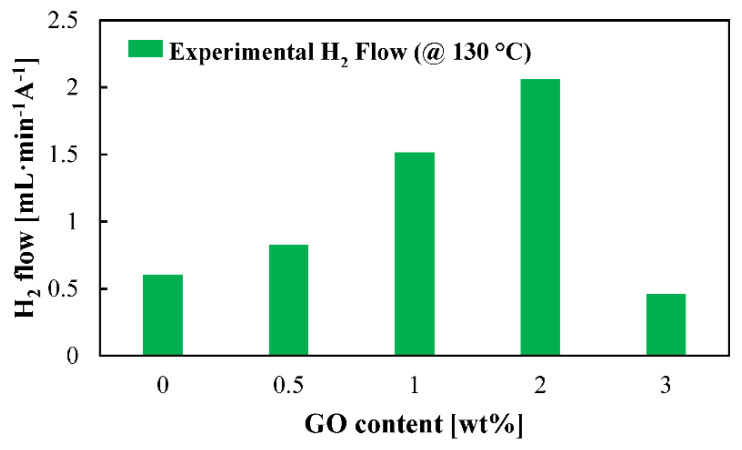
Hydrogen production rate at 130 °C for the studied membranes. Measurements carried out at a cell voltage of 0.6 V.

For the first time, actual H_2_ production is shown for this application (Figure 6) for the five studied membranes at high temperature. Superior H_2_ production rates are obtained for the composite membranes. A clear benefit of introducing graphene oxide as organic filler to the PBI membrane is observed. The hydrogen production will be influenced by the proton transport which, as can be observed in Figure 4, has its minimum values for the cases of the standard and 3 wt % composite membrane. However, this does not explain such a big difference between the 0.5 wt % and the 2 wt % composite membranes. The explanation for this could be explained in terms of crossover. Increasing the filler concentration would increase the tortuosity of the membrane (Figure 1); therefore, the SO_2_ would have more difficulties to cross the membrane. Furthermore, crossover paths are blocked due to GO particles. In this case, the reduced water affinity of the composite membranes toward water demonstrated by the contact angle also explains why more hydrogen is obtained. The reduced water and SO_2_ transport means that side reactions which consume hydrogen, as shown in Equation (7) [50,51], will not occur or their reaction rates will be much lower. 

For the sulfuric acid production rate (Figure S4), the composite membranes with GO content of 0.5, 1, and 2 wt % show the best results, reaching an optimum in production for the composite membrane with 1 wt % of GO. As observed in Figure 5, the composite membrane with 1 wt % of GO shows slightly higher current density values, which explains the small variation in H_2_SO_4_ production. Nevertheless, the results are similar for those three cases. The standard and the 3 wt % composite membrane are the ones with worse rates. One factor that could explain this behavior could be that this membrane reported the lowest proton conductivity, thus showing an overall worse performance.

(7) $${\mathrm{SO}}_{2}+3H_{2}\;\to H_{2}S+2H_{2}O$$

## 4. Conclusions

PBI composite membranes with well-dispersed graphene oxide (GO) were successfully prepared according to the SEM and XRD analysis. The SEM analysis and contact angles showed that the PBI/GO composite membranes were uniform and homogeneous. Membranes with GO content lower than 3 wt % exhibited higher proton conductivities than the standard one. Moreover, the composite-based PBI/GO membranes achieved higher chemical resistant and phosphoric acid retention with respect to the standard PBI membrane.

Furthermore, the PBI/GO composite membranes were tested in an electrolysis cell and operated at a very high temperature: 130 °C. The membranes with 1 or 2 wt % of GO showed a superior performance (in terms of hydrogen production) compared to the rest of the PBI-based membranes tested under the same operation conditions, which makes them suitable to be used for the SO_2_-depolarized electrolysis for hydrogen production.

## Figures and Tables

**Table 1:** Values of the thickness increase, ADL, and phosphoric acid retention of the studied PBI membranes.

Membrane	Thickness Increase [%]	Doping Level	Acid Retention [%]
Standard	106.7	11.4	22.5
0.5 wt % GO	116.7	12.3	44.2
1 wt % GO	120.6	12.8	52.7
2 wt % GO	119.3	12.3	51.3
3 wt % GO	111.7	11.8	60.2

## Supplementary Materials

The following supporting information can be downloaded at https://www.mdpi.com/article/10.3390/membranes12020116/s1, Figure S1: Experimental Set-up; Figure S2: FTIR spectra for the studied membranes; Figure S3: Persulfate chemical oxidation test performed for the studied membranes; Figure S4: Sulfuric acid rate at 130 °C for the studied membranes; Table S1: SO_2_ depolarized results found in literature.
