# Supplementary material for: Characterization of PBI/Graphene Oxide Composite Membranes for the SO2 Depolarized Electrolysis at High Temperature
Source: Membranes (Basel). 2022 Jan 20;12(2):116. doi: 10.3390/membranes12020116 (PMC8875161; doi:10.3390/membranes12020116)
Supplement: Supplementary file 1 [file membranes-12-00116-s001.zip › membranes-1544324-supplementary.pdf]

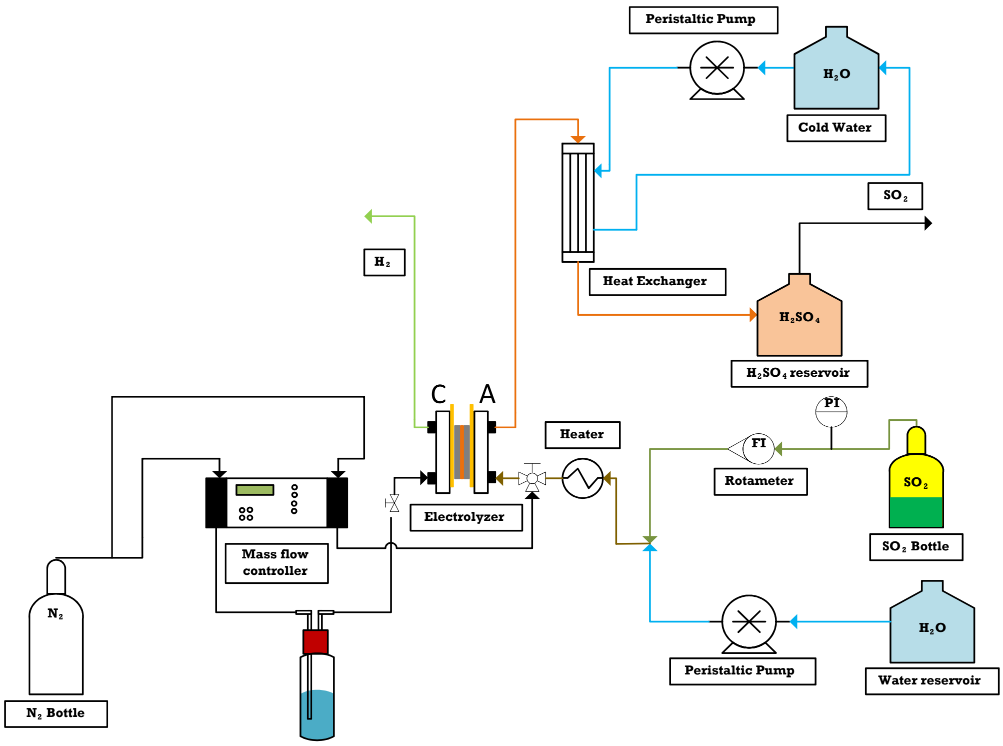

Figure S1. Experimental set-up

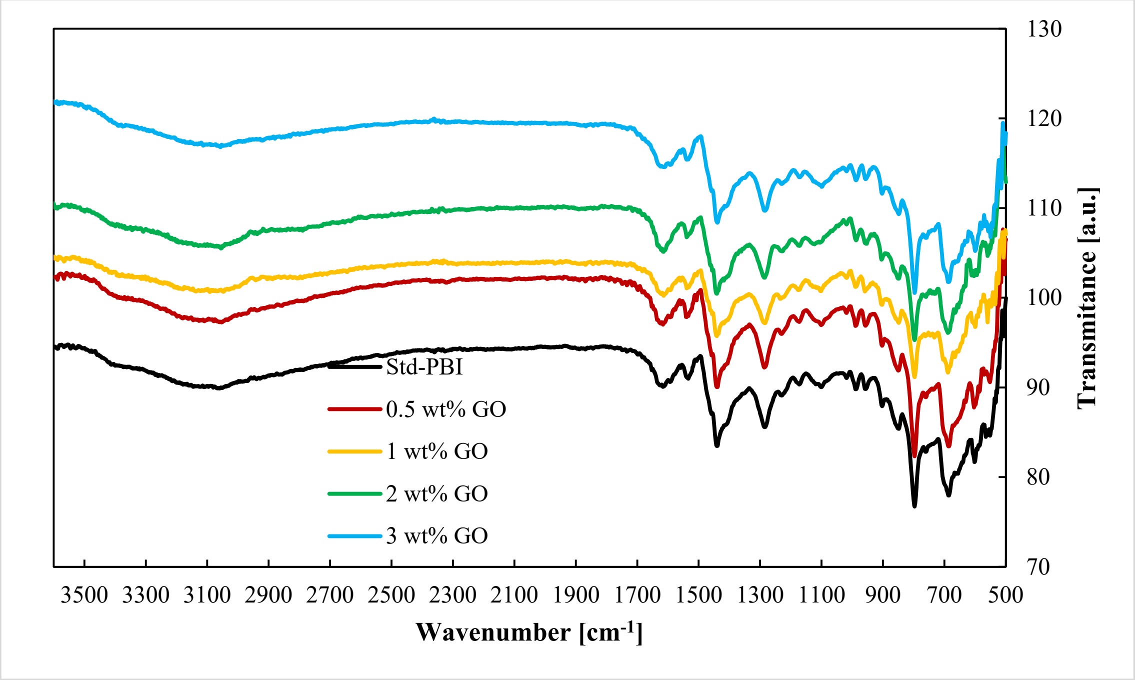

Figure S2. FTIR spectra for the studied membranes

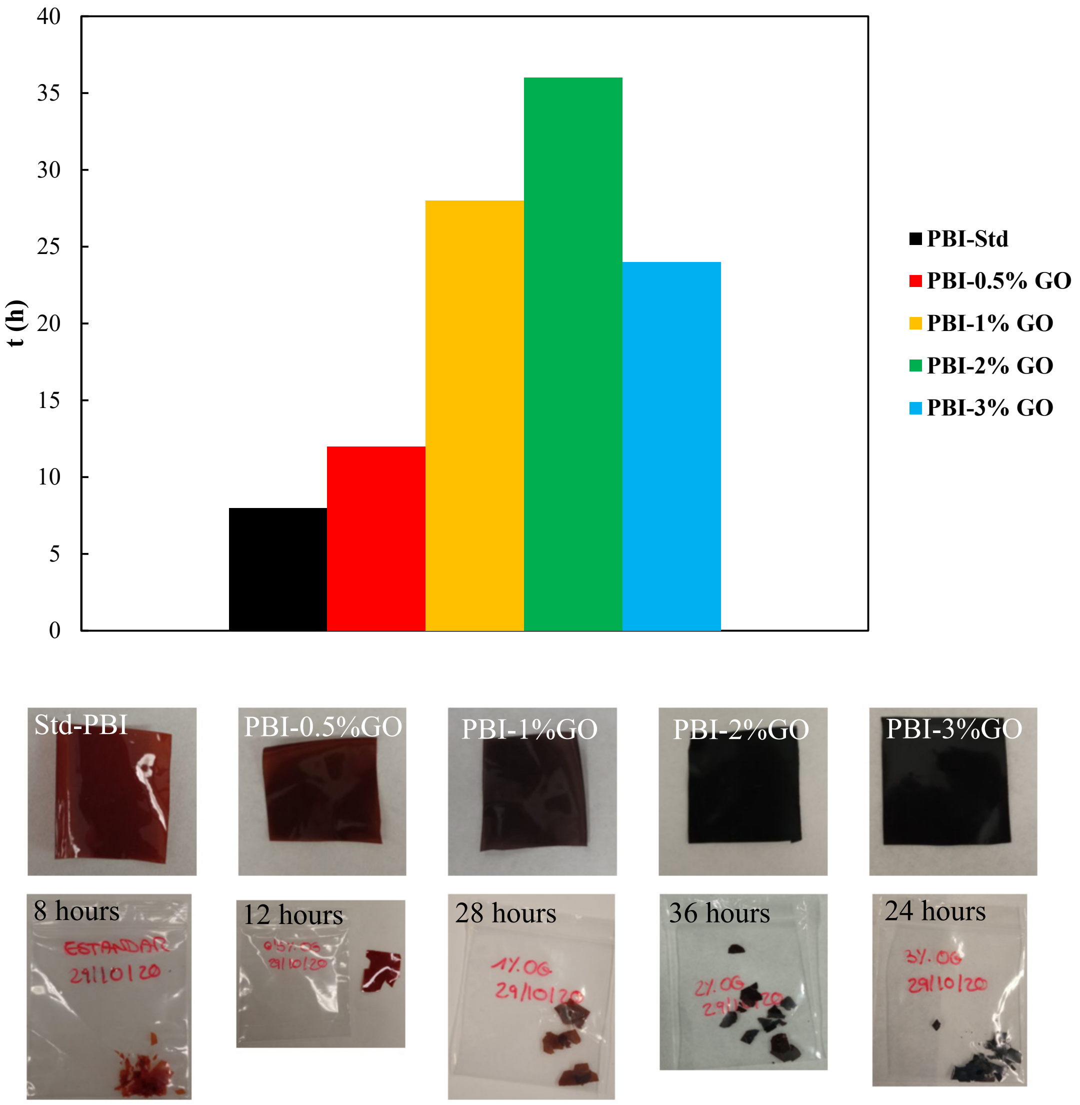

Figure S3. Persulfate chemical oxidation test performed for the studied membranes. Photographs of the membrane before and after the test and the time at which they broke into pieces 1M H_2SO_4; 500ppm $Na_2S_2O_8$; 80 °C

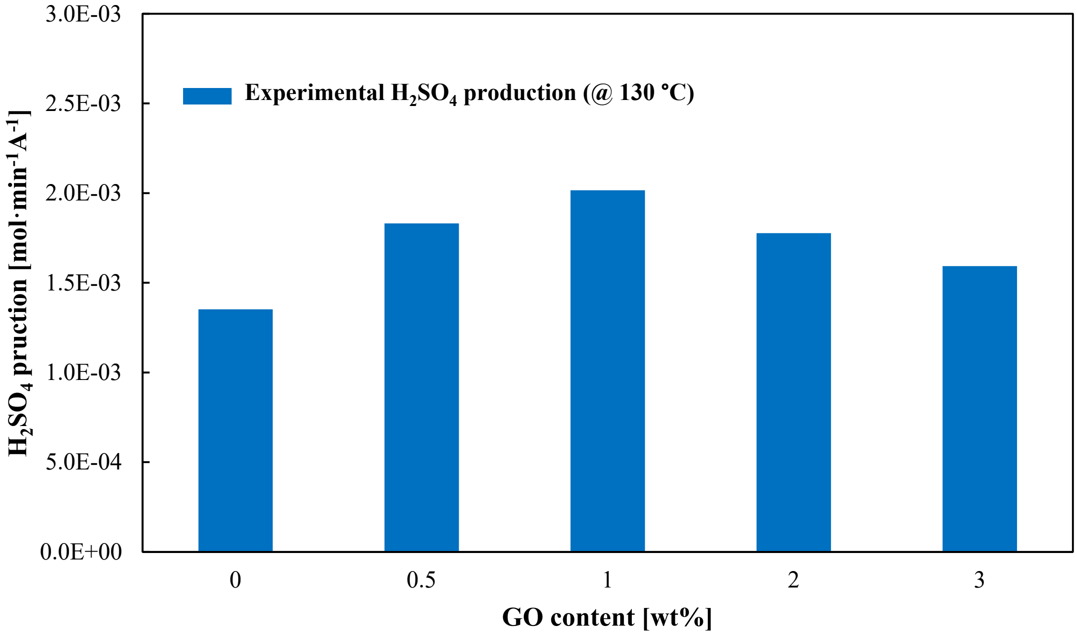

Figure S4. Sulfuric acid rate at 130 °C for the studied membranes. Measurements carried out at a cell voltage of 0.6 V.

Table S1. SO_2 depolarized results found in literature

Reference	Membrane	Voltage [V]	Temperature [°C]	Current Density [mA·cm^{-2}]
[1]	PBI	0.75	80	0.3
[2]	PBI blend	0.85	80	0.3
[3]	Nafion 117	1	80	0.2
[4]	Nafion 115	0.85	80	0.2
This work	PBI-GO (1 wt%)	0.8	130	0.2

1. Peach, R.; Krieg, H.M.; Krüger, A.J.; Bessarabov, D.; Kerres, J. PBI-blended Membrane Evaluated in High Temperature SO2 Electrolyzer. *ECS Trans.* **2018**, *85*, 21–28.

2. Peach, R.; Krieg, H.M.; Krüger, A.J.; Rossouw, J.J.C.; Bessarabov, D.; Kerres, J. Novel cross-linked partially fluorinated and non-fluorinated polyaromatic PBI-containing blend membranes for SO2electrolysis. *Int. J. Hydrogen Energy* **2016**, *41*, 11868–11883, doi:10.1016/j.ijhydene.2016.05.246.

3. Krüger, A.J.; Krieg, H.M.; Grigoriev, S.A.; Bessarabov, D. Various operating methods and parameters for SO2 electrolysis. *Energy Sci. Eng.* **2015**, *3*, 468–480, doi:10.1002/ese3.80.

4. Peach, R.; Krieg, H.M.; Krüger, A.J.; Van Der Westhuizen, D.; Bessarabov, D.; Kerres, J. Comparison of ionically and ionical-covalently cross-linked polyaromatic membranes for SO2 electrolysis. *Int. J. Hydrogen Energy* **2014**, *39*, 28–40, doi:10.1016/j.ijhydene.2013.10.023.
